# Preparation of Sulfonated Poly(arylene ether nitrile)-Based Adsorbent as a Highly Selective and Efficient Adsorbent for Cationic Dyes

**DOI:** 10.3390/polym11010032

**Published:** 2018-12-26

**Authors:** Xuefei Zhou, Penglun Zheng, Lingling Wang, Xiaobo Liu

**Affiliations:** Research Branch of Advanced Functional Materials, School of Materials and Energy, University of Electronic Science and Technology of China, Chengdu 61173, China; zhouxuefei0@hotmail.com (X.Z.); 18482179228@163.com (P.Z.); wangll@std.uestc.edu.cn (L.W.)

**Keywords:** sulfonated poly(arylene ether nitrile), aluminium ions, crosslinking, selective adsorption, cationic dyes

## Abstract

In this work, a highly selective and efficient polymer adsorbent inspired by a water-soluble sulfonated poly(arylene ether nitrile) (SPEN) was successfully synthesized. Due to the distinct structure of functional carboxyl, sulfonic acid and rigid benzene rings, a facile aluminium (III) ions crosslinking method was employed to fabricate the SPEN-based adsorbents (SPEN-Al). Among the three adsorbents, SPEN-Al-2 exhibited superior adsorption capacities with uniform morphology. Subsequently, the SPEN-Al-2 was selected as the adsorbent for three cationic dyes (rhodamine B (Rh B), neutral red (NR), methylene blue (MB)) and three anionic dyes (orange G (OG), methyl orange (MO), acid fuchsin (AF)), respectively, demonstrating that the adsorbent possessing excellent selectivity toward cationic dyes. Moreover, the dye’s adsorption selectivity of SPEN-Al-2 was further certificated in a binary cationic-anionic dyes mixtures (MB/OG and MB/MO) system. Taking MB as a dye model, a series of factors (contact time, concentration, temperature and pH) and adsorption models were systematically investigated in dye adsorption experiments. Results indicated that the adsorption was endothermic and the maximum adsorption capacity of SPEN-Al-2 could reach up to 877.5 mg/g; pseudo-second-model and Langmuir model were fitted to the adsorption kinetics and equilibrium isotherm, respectively, manifesting that SPEN-Al adsorbent was promising in the dyes removing field.

## 1. Introduction

In modern life, people have been accustomed to the world with gorgeous colors, which subsequently drive a steady growing quantity of dye industries varied in different fields such as textile, plastics, printing, food and leather industries [1,2]. However, the discharge of dye effluents into an aqueous ecosystem without prior treatment has brought about serious damages to the environment and human health. Though the polycyclic aromatic, heterocyclic structures in dyes contribute to their high stability to light, heat and catalytic agent, they also increase the toxicity and disposal difficulties of dyestuff wastewater [3,4,5]. Moreover, the co-existence of varied dyes in wastewater has made the treatment more intractable. Therefore, simple and effective dye treatment methods are in great demand to relieve environmental pressure. Numerous methods including ion exchange [6], membrane filtration [7], chemical coagulation/flocculation [8], microbial degradation [9], catalytic reduction [10], etc., have been developed for the treatment of dye-contaminated wastewater. However, these methods always suffer from high cost, complicated operation and generation of potential toxic byproduct [11]. By comparation, the adsorption method exhibited great strengths for wastewater treatment owing to its low energy consumption, high efficiency and ease of operation [12,13]. Moreover, the adsorption route will not produce toxic byproducts, for it is an accumulation process that occurs at the liquid–solid interface or gas–solid interface [14]. Generally, the adsorption includes two approaches, namely physisorption and chemisorption [15]. The physisorption is known as van der Waals adsorption, which is induced by the weak intermolecular forces of adsorbent and adsorbate, including van der Waals forces, hydrogen bonds, π–π interaction, etc. [16,17]. However, physisorption not only requires an adsorbent with a specific surface morphology and porosity but also it needs to be unstable or unselective to dyes. For the chemisorption, it refers to the electrons exchange or transfer between adsorbent and adsorbate surfaces, resulting in the formation of stable chemical bonds between adsorbent and adsorbate [18,19] with high adsorption efficiency. As a result, polymer-based adsorbents have drawn people’s attention, since it is accessible to regulate their specific surface morphology and their functional groups (such as –COOH, –SO_3_H, –NH_2_, –OH etc.), which is crucial for the adsorbent to interact with the targeted adsorbates in the dye removal process [20,21,22].

Poly(arylene ether nitrile) (PEN) is a kind of functional polymer that possesses a rigid aromatic backbone and adjustable side chains, and is famous for its excellent mechanical strength and light and heat stability [23]. After several decades of exploration, the nucleophilic substitution mechanism has developed to be the most mature strategy to synthesize a series of targeted PEN. Typically, a kind of poly(arylene ether nitrile)-containing pendant sulfonic acid group was named as sulfonated poly(arylene ether nitrile) (SPEN), and it has high water adsorption and ionization ability, as well as being considered to be a promising adsorbent candidate for the disposal of dyes wastewater [24]. It has been realized that the higher sulfonation degree is beneficial to ionization and adsorption of SPEN, while it may also result in excessive swelling or dissolution in aqueous solution [25]. Therefore, it was expected to develop a kind of highly efficient SPEN-based adsorbent that is applicable in an everchanging environment. Metallic crosslinking is a widely-accepted method to conquer these defects of SPEN, which are especially adaptable to polymeric adsorbent-containing functional ligands, such as –COOH, –OH [26,27,28]. In addition, the crosslinkers usually come from multivalent metal ions, such as Ca (II), Al (III), Fe (III), Zr (IV) [29,30]. For example, Assia Benhouria et al. have immobilized the alginate, bentonite and activated carbon together with Ca (II) ions and realized improved cationic dye removal efficiency according to the crosslinking mechanism [31].

In this work, a kind of SPEN-Al adsorbent was successfully prepared using a facile aluminium (III) (Al^3+^) ions crosslinking method on the basis of a water-soluble sulfonated poly(arylene ether nitrile) (SPEN). The SPEN-Al-2 was certificated to possess the most uniform morphology and optimal dye-removing ability among the three SPEN-Al adsorbents, and it exhibited highly selective adsorption to cationic dyes, especially for methylene blue. Even for the binary dye mixtures that simultaneously contain cationic (methylene blue) and anionic (orange G or methyl orange) dyes, the selective adsorption ability of SPEN-Al for cationic dyes was still outstanding. Furthermore, a series of factors, including contact time, concentration, temperature and pH, in the dye-removing process were conducted with the methylene blue (MB) as the dyes adsorption model. The results ascertained the equilibrium, kinetics and thermodynamics of the adsorption process, suggesting the SPEN-based adsorbent was highly efficient in selective adsorption for cationic dyes.

## 2. Materials and Methods

### 2.1. Materials

2,6-difluorobenzonitrile (DFBN) and potassium hydroquinone sulfonic acid potassium salt (SHQ) were supplied by Sigma Aldrich (Shanghai, China). Ethanol, phenolphthalein (PP), zinc (Zn), sodium hydroxide (NaOH, AR), aluminium chloride hexahydrate (AlCl_3_·6H_2_O), N-methyl pyrrolidone (NMP, AR), toluene and tetrahydrofuran (THF), sodium dodecyl sulfate (SDS), dichloromethane (CH_2_Cl_2_) and potassium carbonate (K_2_CO_3_, AR) were obtained from Chengdu Haihong Chemical Co. (Chengdu, China). Orange G (OG), methyl orange (MO), Acid fuchsin (AF), rhodamine B (Rh B), neutral red (NR) and methylene blue (MB) were received from Sinopharm chemical reagent (Shanghai, China). Phenolphthalin (PPL) was synthesized from phenolphthalein (PP), Zn and NaOH [25].

### 2.2. Synthesis of SPEN

In a typical synthesis procedure, a mixture of SHQ (20.429 g, 0.0896 mol), PPL (12.223 g, 0.0384 mol), DFBN (17.792 g, 0.128 mol), NMP (65 mL) were added to the three-necked flask, followed by adding a catalyst of K_2_CO_3_ (30.515 g) and dehydrating thr agent of toluene (25 mL), respectively. After a moderate mixing, the mixture was heated to 145 °C and kept for 3 h to remove the generated water, and then the temperature was gradually heated to 155, 165, 175, and 180 °C separately and maintained for 1 h [32]. Furthermore, the raw product was precipitated into ethanol, then it was washed with hot alcohol and deionized water to remove the unreacted reagents and solvent. The final product was dried in a vacuum oven at 80 °C for 24 h.

### 2.3. Preparation of SPEN-Al Adsorbents

The polymer adsorbents were prepared on the basis of our previous work with a slight modification [32]. Briefly, a given concentration of Al^3+^ was firstly prepared in 10 mL aqueous solution and then before being injected into the 20 mL SDS aqueous solution, 118 mg SPEN was added into 1 mL CH_2_Cl_2_ in companion with THF, then the two solutions were mixed together under vigorous stirring in a vial. After the continuous stirring for 24 h, the evaporation of CH_2_Cl_2_ and THF helped the crosslinking between Al^3+^ and SPEN (SPEN-Al). The resultant polymeric adsorbent was washed with deionized water three times to remove the unreacted polymer and ions solution, and then the product was dried in vacuum oven at 60 °C for 48 h. For the concentration of Al^3+^ varied from 0.05, 0.10 to 0.15 M (5, 10, 15 wt %, weight ratio of AlCl_3_·6H_2_O/SPEN), the obtained SPEN-Al were denoted as SPEN-Al-1, SPEN-Al-2, SPEN-Al-3 and their yields were calculated to be 42.3%, 92.7%, 94.8%, respectively. The diameter of irregular SPEN-Al-1 ranged from 50 to 250 nm and the obtained uniform SPEN-Al-2 possessed an average size of 80 nm scale, while a crosslinked net was obtained in SPEN-Al-3.

### 2.4. Adsorption Experiments

The batch adsorption experiments were conducted in a thermostat water bath with a magnetic stirrer. Typically, 10 mg of SPEN-Al adsorbent was mixed with 40 mL of dye solution in a 50 mL vial, which then proceed the adsorption under continuous stirring at certain concentration of dye solution pH and temperature, etc. The adsorption performance of SPEN-Al toward anionic dyes (OG, MO, AF) and cationic dyes (Rh B, NR, MB) was firstly evaluated in neutral condition at 298.15 K with the dye concentration of 100 mg/L. At certain time intervals, the supernatants were centrifuged at 10,000 rpm for 3 min and then analyzed by a ultraviolet-visible spectrophotometer (the maximum adsorption wavelength of OG, MO, AF, Rh B, NR and MB are located at ca. 475, 464, 547, 554, 532, and 664 nm). Similarly, the binary dye mixtures containing cationic MB (40 mg/L, 20 mL) and anionic OG (40 mg/L, 20 mL) or MO (40 mg/L, 20 mL) were prepared, then SPEN-Al adsorbent was added in the mixture for further adsorption in the same condition as above. The dye removal efficiency (*R*), instantaneous adsorption capacity (*q_t_*) and equilibrium adsorption capacity (*q_e_*) were calculated using the following Equations (1)–(3) [33]:

(1) $$R=\left(\frac{C_{o}-C_{e}}{C_{o}}\right)\times 100$$

(2) $$q_{t}=\left(\frac{C_{o}-C_{t}}{m}\right)\times V$$

(3) $$q_{e}=\left(\frac{C_{o}-C_{e}}{m}\right)\times V$$

### 2.5. Characterization

The ultraviolet-visible (UV-vis) absorption spectra of SPEN and all the dyes in aqueous solution were detected with a UV-vis spectrophotometer (TU-1810, Persee, Beijing, China). The chemical structure of SPEN was recorded using a Bruker AV II-400 spectrometer, and the 1H NMR (400 MHz) chemical shifts were measured relative to DMSO-d6 (H:d = 2.50 ppm) as the internal references. Fourier transform infrared (FT-IR) spectra of SPEN were obtained by a Shimadzu 8400S FTIR spectrometer. TGA-Q50 (TA Instruments, Newcastle, DE, USA) were involved in the thermal stability analysis of adsorbents, at a heating rate of 20 °C min^−1^ under nitrogen flowing. Gel permeation chromatography (GPC) of SPEN was detected by Waters Breeze 2 HPLC system (Waters corporation, Milford, CT, USA), using DMF as eluent and poly(methyl methacrylate) as the standard. Morphology and microstructures were characterized by a scanning electron microscope (SEM) (JSM-6490LV, JEOL, Akishima, Japan). Zeta potentials were measured in aqueous solution using a Zeta PALS analyzer (Brookhaven Instruments Corporation, New York, NY, USA). A thermostat water bath (HJ-4B) and pH detector (AH 5201) were involved in the dyes removal experiments. The specific surface area of SPEN-Al was measured on NOVA 4000e adsorption apparatus (Quantachrome Instruments, Boynton Beach, FL, USA) and calculated by the Brunauer-Emmett-Teller (BET) method.

## 3. Results and Discussion

### 3.1. Preparation of SPEN

**Figure 1:**
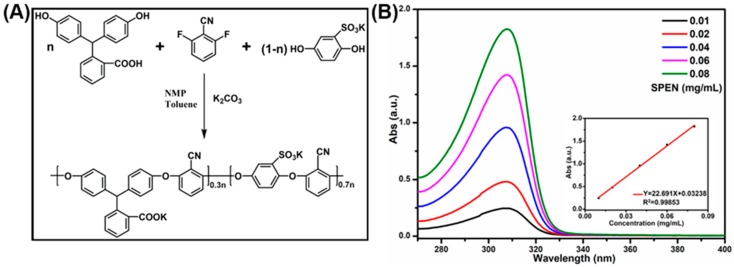
The synthesis route of SPEN and the UV-Vis absorption spectra of SPEN in aqueous solution at different concentrations.

The synthesis of SPEN was based on nucleophilic substitution mechanism and the route was displayed in Figure 1A. The chemical structure of purified SPEN characterized with 1H NMR (DMSO-d6, 400 MHz) indicated that the characteristic peak at 6.69 ppm belongs to tertiary hydrogen atoms in PPL; the hydrogen peaks on benzene rings range from 6.4 to 7.8 ppm, as shown in Figure S1. The functional groups of SPEN were further characterized with FTIR (KBr, cm^−1^): 2230 (C≡N), 1716 (–COO–), 1585–1454 (C=C of Ar), 1243 (Ar–O–Ar), 1076–1018 (S=O of –SO_3_^−^) (see Figure S2). Moreover, the average molecular weight (Mn) and weight average molecular weight (Mw) were 16303 and 20306 g mol^−1^, with a polydispersity (Mw/Mn) of 1.25. Besides, Figure 1B displayed the UV-Vis absorption spectra of SPEN in aqueous solution. The UV-Vis absorption peaks of SPEN at 308 nm proportionally increased when their concentrations increasing from 0.08 mg/mL to 0.01 g/mL. Moreover, a calibration curve mapping the absorption intensities (Y) and concentrations (X) was calculated as: Y = 22.691X + 0.03238, with a high linear correlation coefficient of 0.99853, suggesting that the pendent carboxyl and sulfonic acid groups endowed SPEN with absolute solubility in the aqueous solution.

### 3.2. Morphology Evolution

**Figure 2:**
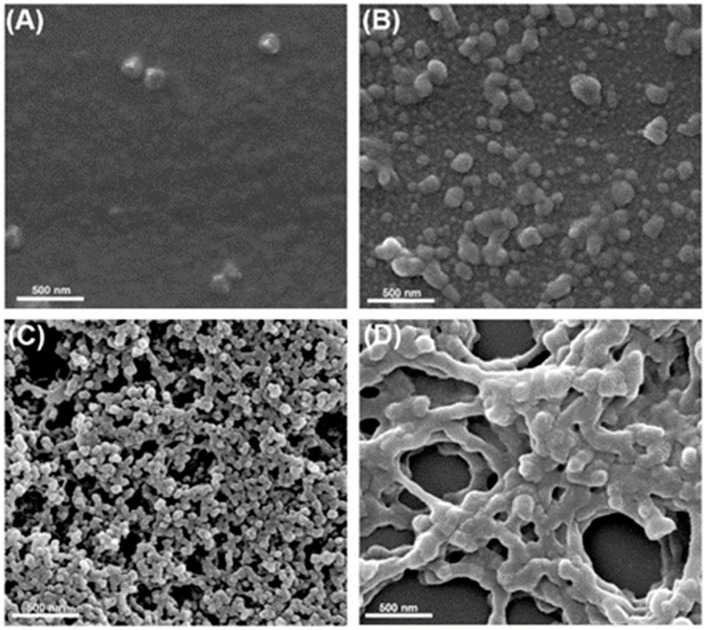
SEM images of SPEN before (**A**) and after crosslinking with Al^3+^ at different concentrations: (**B**) 0.05 M, (**C**) 0.10 M and (**D**) 0.15 M in aqueous solution.

Inspired by the high hydrophilia of sulfonic acid groups, the crosslinking ability of carboxyl and the rigid main skeleton of the benzene ring, the SPEN was considered to be a potential candidate in the dyes removing field. A self-assembling method was then developed to fabricate the SPEN-based adsorbent using the Al^3+^ as a crosslinker. The concentration of Al^3+^ was closely related with the –COOH content in the resulted SPEN adsorbent; three Al^3+^ in the concentration of 0.05, 0.1 and 0.15 M were selected to crosslink SPEN. Based on the molecular weight of the monomeric unit of SPEN (about 366 g/mol) and the concentrations of Al^3+^, the molar ratio of Al^3+^ and –COOH were figured out to be 1:4.3, 1:2.15 and 1:1.433, respectively. The crosslinked SPEN-Al adsorbents were firstly characterized by a scanning electron microscope; the morphologic change of SPEN is shown in Figure 2. It is observed in Figure 2A that the raw SPEN without any Al^3+^ exhibits a flat interface. When the Al^3+^ with a concentration of 0.05 M was involved in the system, the irregular SPEN-Al-1 with a diameter ranging from 50 to 250 nm is observed in Figure 2B. The ununiform structure may be attributed to the heterogeneous reaction between SPEN and Al^3+^, since the crosslinker was uncapable to interact with too much carboxyl groups simultaneously [34]. Besides, the low yield of SPEN-Al was calculated to be as low as 42.3%, which was ascribed to the removal of a large proportion of un-crosslinked SPEN in the purification process. For comparison, the homogeneous SPEN-Al-2 adsorbent with an interconnected particle of 80 nm was achieved when the concentration of Al^3+^ increased to 0.10 M, as shown in Figure 2C. The yield of SPEN-Al-2 reached up to 92.7%, indicating the high crosslinking efficiency between SPEN and Al^3+^. When the concentration of the crosslinker continue increased to 0.15 M, the obtained SPEN-Al-3 in Figure 2D displayed a crosslinked net with many spherical adsorbents precipitated on the crosslinked skeleton, and the yield of SPEN-Al-3 was calculated to be 94.8 %. It was speculated that the excessive crosslinker connected the initially formed SPEN-Al adsorbent and evolved into the polymer nets with the sphere adsorbent immobilized on it. The evolution of microscopic morphology confirmed that the Al^3+^ was capable of adjusting the morphologies of SPEN-Al.

### 3.3. Thermal Stability

**Figure 3:**
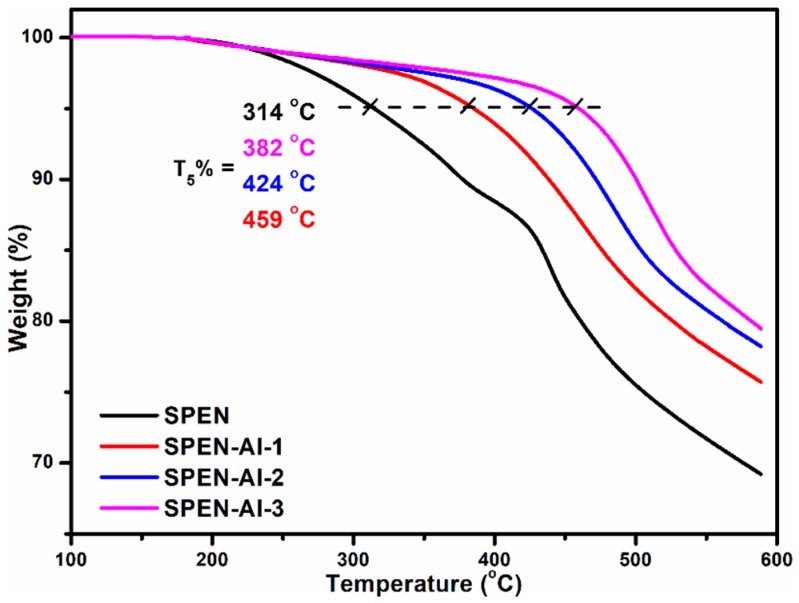
TGA curves of raw SPEN and SPEN-Al induced by Al^3+^ in different concentrations.

The thermogravimetric characterizations were carried out to evaluate the stability of the as-prepared adsorbents. As the TGA curves show in Figure 3, the 5% weight loss temperature of raw SPEN was located at 314 °C, which was mainly attributed to the decomposition of carboxyl and sulfonic acid [23]. In addition, the obvious weight loss of SPEN at 420 °C corresponded to the decomposing of the benzene ring in the backbone. By comparison, the 5% weight loss temperatures of SPEN-Al continuously increased from 382 to 459 °C along with the concentrations of the crosslinker increasing from 0.05 to 0.15 M, indicating that the crosslinking with Al^3+^ would improve the thermal stability of SPEN-based adsorbents. The improved thermal stability of SPEN-Al-3 also implied that the SPEN-Al-1 and SPEN-Al-2 still reserved unreacted functional groups, which would work in the subsequent dyes adsorption.

### 3.4. Selective Adsorption for Dyes

**Figure 4:**
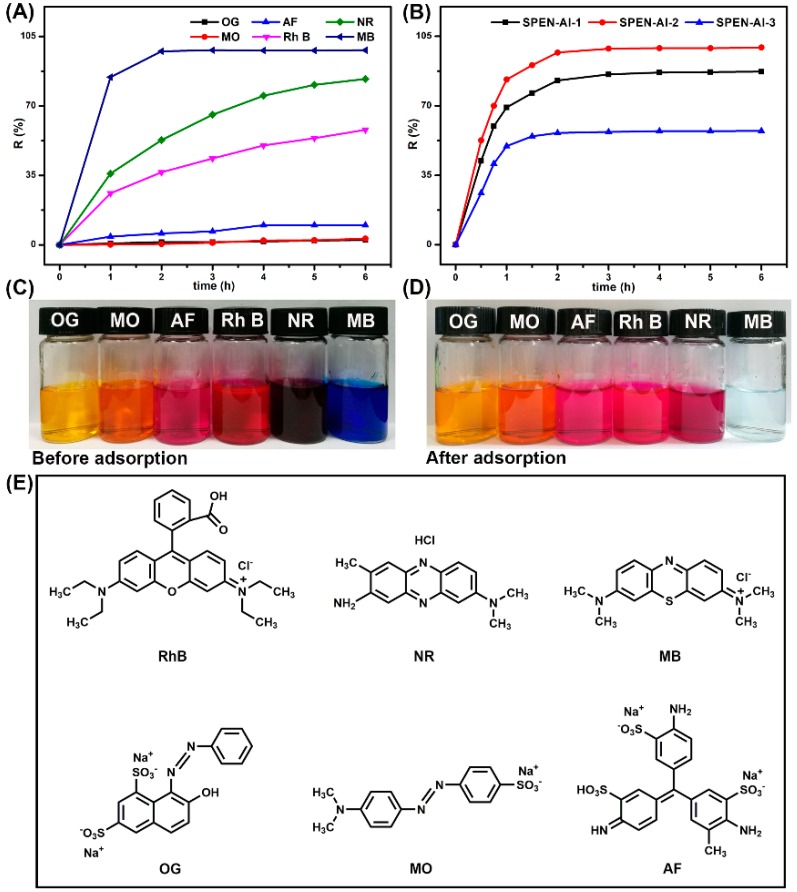
Effect of contact time on the removal efficiency of six dyes by the certain SPEN-Al-2 adsorbent (**A**) and the removal efficiency of MB by three different SPEN-Al adsorbents (**B**). Digital photos of the organic dyes in aqueous solutions before (**C**) and after (**D**) adsorption by SPEN-Al-2. Chemical structures of organic dyes (Rh B, NR, MB, OG, MO and AF) selected for adsorbent (**E**).

Next, three cationic dyes (Rh B, NR, MB) and three anionic dyes (OG, MO, AF) of the same concentration of 100 mg/L were applied to evaluate the adsorption performance of SPEN-Al at 298.15 K, respectively. The results shown in Figure 4A display that the dye removal efficiency for cationic dyes of Rh B, NR and MB have reached 57.96%, 83.61% and 98.08% within 6 h, respectively. The different dye removal efficiencies were closely related to the structure of the dyes. As shown in Figure 4D, MB was a kind of phenothiazine salt containing dimethylamino, which would ionized in an alkaline type in aqueous solution; whereas the alkalescent NR was a kind of phenazine hydrochloride, whose ionization was restricted due to the presence of HCl. Though the Rh B was also a cationic dye due to the ionization of diethylin, the coexisting carboxyl prefered to repel the anionic SPEN-Al owing to like charges repelling each other. Furthermore, the molecular volume of the three dyes may also bring out the differences in their adsorption capacities. A smaller molecular volume would accelerate the dye’s mobility and then promote it to interact with the adsorbent. In comparison with MB, NR and Rh B, the largest volume of Rh B was consistent with its lowest adsorption efficiency. Therefore, the different adsorption selectivity of the SPEN-Al adsorbent for MB, Rh B and NR was induced by the ionization ability and molecular volume of dyes, resulting in the SPEN-Al adsorbent showing high selectivity for MB compared to RhB and NR.

The SPEN-Al almost makes no sense to anionic dyes, as their absorbances changed very little as the images show in Figure 4C,D, respectively. Besides, the variations of the absorption intensity in the dye-removal process were detected by UV-vis spectrophotometer, respectively, as shown in Figure S3. The vast adsorption difference for anionic and cationic dyes indicated that the electrostatic interaction may be the main force in the dye’s adsorption process. This is because SPEN-Al adsorbent was negatively charged owing to the ionization of –COOK and –SO_3_K, which made it attractive to cationic dyes and repulsive to anionic dyes. To select the optimal absorbent, three kinds of SPEN-Al were applied in the adsorption for MB (100 mg/L, 40 mL) at 298.15 K, respectively. The results displayed in Figure 4B show that the dyes adsorption efficiency of SPEN-Al-1, SPEN-Al-2 and SPEN-Al-3 were 87.23%, 98.08% and 57.34%, respectively. When it comes to SPEN-Al-1 with a low yield of 42.3%, long-time adsorption would cause its dissolution in dye solution and give rise to low adsorption efficiency. As for SPEN-Al-3, the excessive Al^3+^ would consume a large quantity of the active sites in the adsorbent, resulting in a crosslinked net morphology (Figure 2D) and low dye adsorption efficiency. Obviously, the uniform SPEN-Al-2 which originated from moderate Al^3+^ concentration was optimum for dye adsorption.

**Figure 5:**
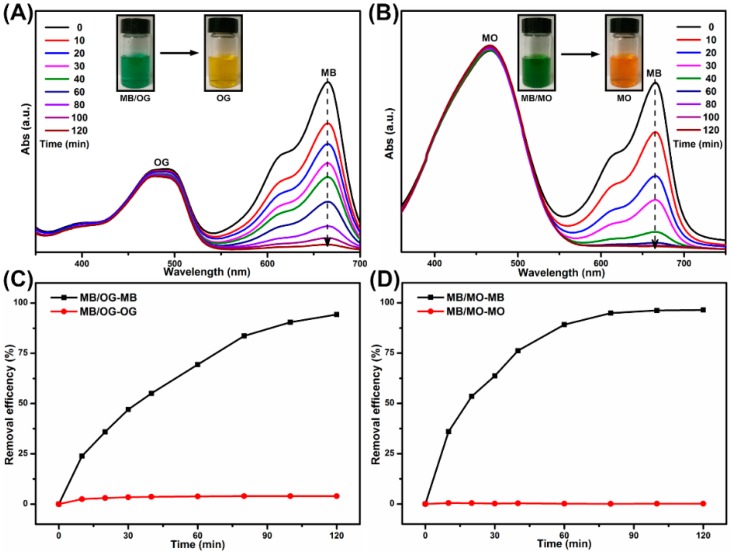
The UV-vis spectra and the corresponding removal efficiency of MB/OG mixture (**A**) (**C**) and MB/MO mixture (**B**) (**D**) onto SPEN-Al-2 at different contact times.

The nitrogen adsorption–desorption isotherm of the SPEN-Al-2 obtained at 77 K is exhibited in Figure S4, which indicated that the specific surface area of the adsorbent was about 4.18 m^2^ g^−1^. The isotherm plot of SPEN-Al-2 was classified to type III with a hysteresis loop in the relative pressure range of 0.9–1.0, suggesting that the physical interaction between the adsorbent and nitrogen was weak. Apart from cationic dyes in the one-component solution, the dyes mixtures (MB/OG and MB/MO) that simultaneously contain cationic dye (MB) and anionic dye (OG or MO) were also prepared to explore the adsorption selectivity of SPEN-Al-2. As shown in Figure 5A,B, the characteristic absorption peak of MB at 664 nm continuously decreased after adding SPEN-Al-2, while the other absorption peaks of OG at 475 nm or MO at 464 nm remained almost unchanged. As the inset shown in Figure 5A, the color of the mixture dyes containing MB/OG varied from turquoise to orange yellow while the MB was not observed. The color change indicated that the MB was removed by SPEN-Al-2 but the MO was left. Similarly, the inset of Figure 5B also presents the color changes of MB/MO dye mixture from olive drab to golden yellow with the help of SPEN-Al-2, suggesting the selective adsorption of SPEN-Al-2 to cationic MB dye. In addition, the dye removal efficiency of MB in MB/OG and MB/MO dye mixtures were respectively calculated to be 95.4% and 97.5% within 120 min, respectively, as shown in Figure 5C,D. The different dye removing efficiencies in MB/OG and MB/MO systems were highly dependent on the anionic groups of OG and MO, respectively. Comparing the anionic dyes of OG and MO, it was found that an OG molecular has two sulfo groups while a MO molecular only possesses one sulfo group. The stronger repulsive interaction between OG and SPEN-Al in the MB/OG system was considered to hinder the removal of MB more obviously than the repulsive interaction in MB/MO system. These results certified that the SPEN-Al-2 adsorbent maintained adsorption ability to cationic MB dye even in the binary dyes mixture, thus the MB was selected as the dye model to systematically investigate the adsorption behavior of SPEN-Al.

### 3.5. Effect of Initial Solution pH

**Figure 6:**
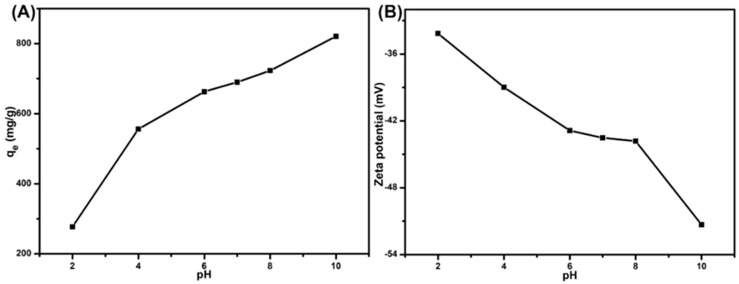
The effect of solution pH on the adsorption of MB onto the SPEN-Al-2 (**A**). Tthe variation of zeta potentials of SPEN-Al-2 versus the solution pH (**B**).

Since the variation of pH was closely related to the ionization of the adsorbent and adsorbate, the effects of pH were investigated to evaluate the adsorption performance of SPEN-Al-2. To study the effect of pH, 10 mg of SPEN-Al was mixed with 40 mL of MB (300 mg/L) in the condition of 298.15 K and different initial pH; the contact time was fixed to be 12 h. The initial pH of MB ranging from 2 to 10 was regulated by 1.0 mol/L NaOH and 1.0 mol/L HCl aqueous solution. As shown in Figure 6A, the adsorption capacity presented an intense increase from 690.95 to 821.07 mg/g with the pH of MB solutions having increased from 7 to 10, while it decreased to 277.19 mg/g at a pH of 2. On the other hand, the zeta potential of SPEN-Al-2 dispersed in aqueous solution was detected. Results indicated the SPEN-Al-2 were negatively charged, and the zeta potential presented a decreased trend along with the increase of pH, as shown in Figure 6B, suggesting the high original ionization ability of SPEN-Al-2. The zeta potential manifested that alkaline solutions would contribute to the ionization of MB and activated the positively-charged groups on SPEN-Al-2, which subsequently would enhance the electrostatic interaction between the adsorbent and adsorbate, resulting in an increased adsorption capacity [35]. The acidic environment tended to restrict the ionization of carboxyl radicals and sulfonic radicals of SPEN-Al-2, which would bring in not only the electrostatic repulsion between SPEN-Al-2 and MB but also the competitive adsorption between H^+^ ions and cationic MB, resulting in a decreased adsorption capactity [33].

### 3.6. Adsorption Kinetics

**Figure 7:**
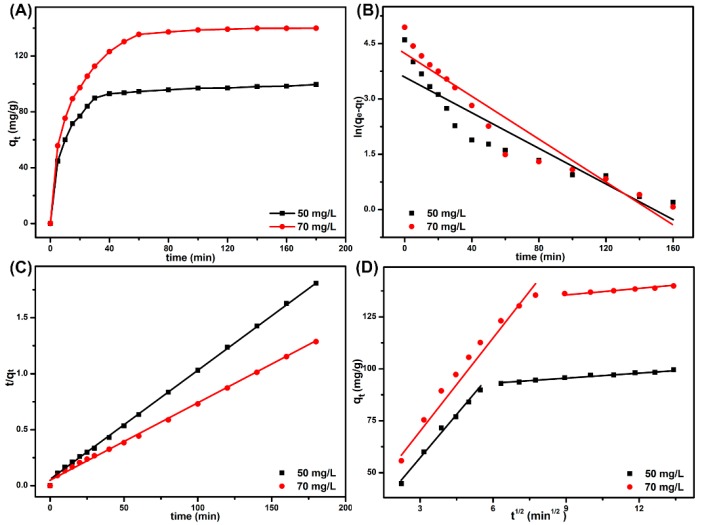
Effect of contact time on MB adsorption capacity (**A**), pseudo-first-order (**B**), pseudo-second-order (**C**) and intraparticle diffusion (**D**) for the adsorption of MB onto SPEN-Al-2.

As the results show in Figure 7A, the SPEN-Al-2 gave increased equilibrium adsorption capacities in two MB solutions (50 and 70 mg/L), since the initial MB concentrations can provide a driving force to overcome the mass transfer resistance of the dye. It was clear that both the two curves presented a fast increase at the initial time and then they slowed down to get the balanced state, reaching the adsorption equilibrium within 180 min. The drastic changes may be closely related with the available sites on the surface of SPEN-Al-2. At the beginning, the MB molecules rapidly occupied the empty sites on SPEN-Al adsorbent, resulting in a drastic increase in the adsorption curves. After the full occupation of valuable sites, the SPEN-Al-2 was not available for any anionic MB, resulting in the platform in the graph. The kinetics of dye adsorption is one of the main preconditions in selecting the operational environment. To elaborate the dye’s adsorption behavior, three conventional models that are denoted as pseudo-first order, pseudo-second order and intraparticle diffusion models have been widely accepted; these formulas are described as following Equations (4) and (5) [12]:

(4) $$q_{t}=q_{e}\left(1-e^{-k_{1}t}\right)$$

(5) $$q_{t}=\frac{k_{2}q_{e}^{2}t}{1+k_{2}q_{e}t}$$

where *k*_1_ (min^−1^) and *k*_2_ (g mg^−1^ min^−1^) are the rate constants of pseudo-first-order and pseudo-second-order adsorption, respectively; *t* (min) is the contact time.

Based on Figure 7A, the corresponding linear regression equations were calculated and displayed in Figure 7B,C, respectively. Moreover, the kinetic parameters of the correlation coefficient (*R*^2^), *k*_1_, *k*_2_ and calculated *q_e_* (cal.) are exhibited in Table 1. It was observed that the linear regression equations from the two MB solutions (50 and 70 mg/L) followed the same adsorption kinetics. Taking the adsorption of 50 mg/L MB for example, the *R*^2^ (0.8543) calculated from pseudo-first-order was much lower than *R*^2^ (0.9992) of pseudo-second-order. Moreover, the adsorption capacity calculated from the pseudo-second-order (102.04 mg/g) was close to the experimental results (99.539 mg/g). In summary, the adsorption of MB onto SPEN-Al-2 belonged to pseudo-second-order, which corresponds with the assumption that the adsorption rate was mainly controlled by chemical adsorption.

Generally, the adsorption occurred in several steps; the process was usually studied using the intraparticle diffusion model, as the following Equation (6) exhibits [14]:

(6) $$q_{t}=k_{i}t^{0.5}+C$$

where *k_i_* (mg g^−1^ min^−0.5^) is the intraparticle diffusion rate constant and *C* (mg g^−1^) is a constant that can be used to evaluate the effect of boundary layer thickness. Similarly, the linear regression equations were also calculated on the basis of Figure 7A. As for the adsorption of MB with a concentration of 50 mg/L, the two separated linear equations in Figure 7D suggested that the adsorption of MB onto SPEN-Al-2 needs two steps. The first step of adsorption belonged to film diffusion, namely the MB transport from the aqueous solution to the surface of SPEN-Al-2. The second step was the interparticle diffusion, which was attributed to the rough surface and interior of SPEN-Al-2. That the value of k_i1_ was much higher than k_i2_ indicated the intraparticle diffusion was a gradual process. Moreover, the high value of C implied that the intraparticle diffusion was not the rate-limiting step and the film diffusion was important in the MB adsorption process.

### 3.7. Adsorption Isotherm

**Figure 8:**
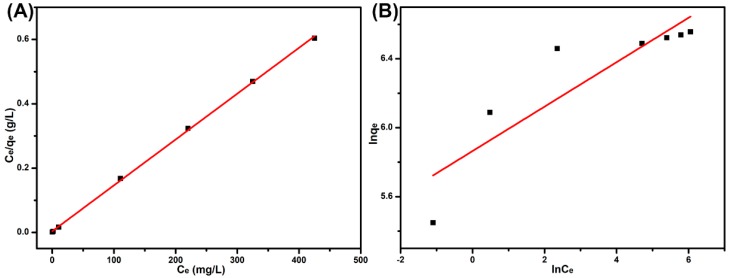
Langmuir isotherm (**A**) and Freundlich isotherm (**B**) for the adsorption of MB onto SPEN-Al-2.

The adsorption isotherm is another significant model that helps to explain the interacting behavior between the adsorbent and adsorbate. The experiments were performed in different concentrations (100–700 mg/L) of MB solution at 298.15 K in a neutral environment. The experimental equilibrium data of MB adsorption onto SPEN-Al-2 are fitted to Langmuir (Figure 8A) and Freundlich (Figure 8B) models, which are defined as the following Equations (7) and (8) [36]:

(7) $$\frac{C_{e}}{q_{t}}=\frac{1}{K_{L}q_{m}}+\frac{C_{e}}{q_{m}}\;$$

(8) $$\ln q_{e}=\ln K_{F}+\frac{1}{n}\ln C_{e}$$

where *K_L_* (L/mg) is the Langmuir adsorption equilibrium constant and *q_m_* is the maximum adsorption capacity (mg/g). *K_F_* and n are Freundlich constants. The Langmuir model is applicable to monolayer adsorption with homogeneous active sites on the surface of the adsorbent [37]. To the contrary, the Freundlich adsorption model is suitable for heterogeneous adsorption, because the available sites on the adsorbent are inconsistent [38]. As the isotherms in Figure 8 and adsorption isotherm constants in Table 2 show, the adsorption isotherm was fitted to the Langmuir model, because *R*^2^ (0.99952) of the Langmuir model was close to 1 and larger than *R*^2^ (0.75912) of the Freundlich model. In addition, the maximum adsorption capacity (704.28 mg) of SPEN-Al-2 calculated from the Langmuir model was also close to the experimental date (690.95 mg/g), and the molar ratio of adsorbed MB and anion units in SPEN-Al-2 in the equilibrium state was calculated to be 1:1.135. Similarly, on the basis of the maximum adsorption capacity of SPEN-Al-1 (639 mg/g) and SPEN-Al-3 (352 mg/g), the molar ratio of the cationic MB and anion units on SPEN-Al were calculated to be 1:0.763 and 1:1.313, respectively. It is suggested that SPEN-Al-2-containing homogeneous active sites and adsorption follow the Langmuir model. What is more, a separation factor related with the Langmuir model denoted as *R_L_* was applied to evaluate the type of adsorption and the relation was displayed as below [36]:

(9) $$R_{L}=\frac{1}{1+K_{L}C_{o}}$$

With the initial concentration of *Co* (mg/L) and Langmuir constant *K_L_* (L/mg) in mind, the *R_L_* was calculated in the range of 0.02708 to 0.00396, indicating that the MB adsorption on SPEN-Al-2 was favorable. Because the value of *R_L_* represented the isotherm was either irreversible (*R_L_* = 0), favorable (0 < *R_L_* < 1), linear (*R_L_* = 1) or unfavorable (*R_L_* > 1) [36].

### 3.8. Adsorption Thermodynamics

**Figure 9:**
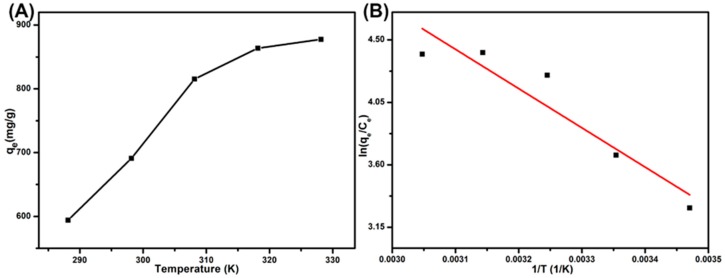
The effect of temperature on the adsorption of MB onto SPEN-Al-2 (**A**) and thermodynamic parameters for the adsorption of MB onto SPEN-Al-2 (**B**).

The thermodynamic analyses were conducted by using 10 mg of SPEN-Al and 40 mL of MB solution (300 mg/L) in neutral condition. Figure 9A presents the different equilibrium adsorption capacities of SPEN-Al-2 to MB at five different temperatures. The adsorption capacity increased from 594.2 mg/g at 288.15 K to 877.5 mg/g at 328.15 K, which implied that the MB adsorption on SPEN-Al-2 was an endothermic reaction. To explore the internal energy changes in the dye adsorption process, the thermodynamic models calculated from Gibbs free energy change (*∆G^o^*), enthalpy change (*∆H^o^*) and entropy change (*∆S^o^*) are defined as the following Equations (10) and (11):

(10) $$∆G^{o}=-RT\ln K_{c}$$

(11) $$\ln K_{c}=-\frac{∆H^{o}}{RT}+\frac{∆S^{o}}{R}$$

where *R* and *T* are the universal gas constant (8.314 J/mol K) and the system temperature (*K*), *K_c_* (*q_e_*/*C_e_*) represent the Langmuir equilibrium constant (L/mol). The thermodynamic model and related parameters are exhibited in Figure 9B and Table 3. Results indicated that the negative value of *∆G^o^* gradually decreased from −7.879 KJ/mol at 288.15 K to −11.998 KJ/mol at 328.15 K, confirming the adsorption process was spontaneous and more favorable at high temperature [39]. The positive value of *∆H^o^* also suggested the adsorption was an endothermic reaction, which is consistent with the result from Figure 9A. In addition, the positive value of *∆S^o^* manifested in the enhanced randomness at the solid–solute interface, for the rising temperature has promoted the mobility of dye molecules and increased the number of active sites in the adsorption process [40].

### 3.9. Adsorption Mechanism

**Figure 10:**
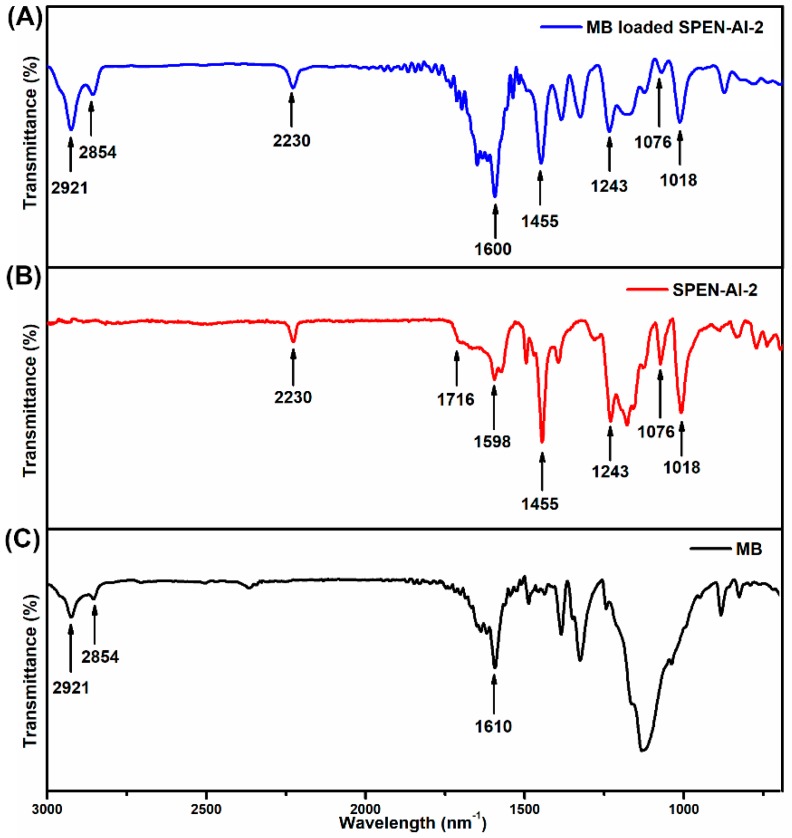
FTIR spectra of MB and SPEN-Al-2 before and after adsorption of MB.

The FTIR spectra of MB, SPEN-Al-2 and MB-loaded SPEN-Al-2 are conducted and studied to gain insight into the adsorption mechanism, as displayed in Figure 10. The characteristic adsorption bands of MB at 2921 and 2854 cm^−1^ belonged to C–H symmetric and asymmetric stretching vibrations of methyl; the band located at 1610 cm^−1^ corresponded to C=C skeletal vibrations of the benzene ring, as shown in Figure 10A. Compared with virgin SPEN in Figure S2, the spectra of SPEN-Al in Figure 10B exhibited a weakened absorption peak of –COO– at 1716 cm^−1^ after crosslinking with Al^3+^. Besides, the FTIR spectra of MB-loaded SPEN-Al-2 demonstrated new absorption bands at 2921 and 2854 cm^−1^, in which the bands were consistent with the C–H symmetric and asymmetric stretching vibrations of methyl on the MB molecule, indicating the MB was indeed adsorbed onto SPEN-Al-2. Moreover, the characteristic absorption band of –COO– at 1716 cm^−1^ disappeared and the S=O at 1076 and 1018 cm^−1^ were weakened, which is owing to the electrostatic interaction between anionic SPEN-Al-2 with cationic MB. Specifically, the negatively-charged –COO– and –SO_3_^−^ on SPEN-Al-2 supplied plenty of active sites for the electrostatic interaction with positively-charged MB. There was also a slight shift of the absorption band of C=C skeletal vibrations varying from 1598 cm^−1^ to 1600 cm^−1^, indicating the weak π–π interaction between SPEN-Al and MB. Therefore, the high selectivity and adsorption capacity of SPEN-Al-2 would be mainly attributed to the electrostatic interaction, π–π interaction and specific surface morphology between SPEN-Al-2 and cationic MB.

## 4. Conclusions

In conclusion, a new polymeric adsorbent was developed by a facile crosslinking method with Al^3+^ and it proved to be a highly selective and efficient adsorbent for cationic dyes. Originating from the crosslinking ability of the pendent carboxyl groups on SPEN, the optimal adsorbent of SPEN-Al-2 prepared using 0.1 M Al^3+^ was found to possess excellent stability and dye-removing ability. Moreover, the SPEN-Al-2 presented high adsorption selectivity to cationic dyes (MB, NR, Rh B), to which selectivity was also applicable in the binary cationic–anionic dye mixtures (MB/OG and MB/MO) system. Results indicated that the higher pH value, concentration or temperature were all in favor of the adsorption for cationic MB, and the maximum adsorption capacity of SPEN-Al-2 towards MB could reach up to 877.5 mg/g at 328.15 K in neutral environment. The high adsorption capacity can be mainly ascribed to the electrostatic interactions and the structure property of SPEN-Al-2 adsorbent.

The kinetic studies demonstrated that the adsorption included two diffusion steps and was fitted to a pseudo-second-order kinetic model. The Langmuir model elaborated that the SPEN-Al-2 have homogeneous active sites, and thermodynamic analysis certified that the MB adsorption was a spontaneous as well as endothermic reaction. It is expected that the series of SPEN-based adsorbents will be explored for dye adsorption from the dye effluents.

## Figures and Tables

**Table 1:** The kinetic parameters of adsorption of MB onto SPEN-Al-2.

C_0_ (mg/L)	Parameters	50	70
Pseudo-first-order	*k*_1_(min^−1^)	0.0244	0.0296
	*q_e_*(cal.)(mg/g)	35.701	69.650
	*q_e_*(exp.)(mg/g)	99.539	139.88
	*R* ^2^	0.8543	0.9166
Pseudo-second-order	*k*_2_(g/mg min)	0.0020	0.0010
	*q_e_*(cal.)(mg/g)	102.04	145.77
	*q_e_*(exp.)(mg/g)	99.539	139.88
	*R* ^2^	0.9992	0.9981
Intraparticle diffusion	*k_i_*_1_	13.704	14.286
	*C*	15.853	30.739
	*R*_1_^2^	0.9890	0.9725
	*k_i_*_2_	0.8832	0.7909
	*C*	87.589	129.03
	*R*_2_^2^	0.9795	0.9811

**Table 2:** Adsorption isotherm constants for the adsorption of MB onto SPEN-Al-2.

Isotherms	Parameters (Temperature = 298.15 K)	
Langmuir	*q_m_* (mg/g)	699.301
	*K_L_*(L/mg)	0.35930
	*R_L_*	0.01373
	*R* ^2^	0.99952
Freundlich	*K_F_*[(mg/g)(L/mg)^1/n^]	15.9420
	*n* ^−1^	0.12896
	*R* ^2^	0.75912

**Table 3:** Thermodynamic constants of the adsorption of MB onto SPEN-Al-2.

*T* (K)	Thermodynamic Parameters			
	ln*K*	*ΔG*^o^ (KJ/mol)	*ΔS*^o^ (J/(mol K)	*ΔH*^o^ (KJ/mol)
288.15	3.289	−7.879	109.78	23.527
298.15	3.669	−9.095		
308.15	4.245	−10.877		
318.15	4.409	−11.662		
328.15	4.398	−11.998		

## Supplementary Materials

The following are available online at http://www.mdpi.com/2073-4360/11/1/32/s1, Figure S1: The 1H NMR of SPEN. Figure S2: The FTIR of SPEN. Figure S3: The UV-Vis spectra of different dyes: orange G (A), methyl orange (B), acid fuchsin (C), rhodamine B (D), neutral red (E) and methylene blue (F) adsorption onto SPEN-Al at specific time intervals, respectively. Figure S4. Nitrogen adsorption–desorption isotherm for the SPEN-Al adsorbent.
