# Supplementary material for: Preparation of Sulfonated Poly(arylene ether nitrile)-Based Adsorbent as a Highly Selective and Efficient Adsorbent for Cationic Dyes
Source: Polymers (Basel). 2018 Dec 26;11(1):32. doi: 10.3390/polym11010032 (PMC6401942; doi:10.3390/polym11010032)
Supplement: Supplementary file 1 [file polymers-11-00032-s001.pdf]

Supplementary information

Preparation of sulfonated poly(arylene ether nitrile)-based adsorbent as highly selective and efficient adsorbent for cationic dyes

Xuefei Zhou, Penglun Zheng, Lingling Wang and Xiaobo Liu*

Research Branch of Advanced Functional Materials, School of Materials and Energy, University of Electronic Science and Technology of China, Chengdu 61173, China; zhouxuefei0@hotmail.com (X.Z.); 18482179228@163.com(P.Z.); wangll@std.uestc.edu.cn(L.W.)

*Correspondence: liuxb@uestc.edu.cn (X.L); Tel.: +86-28-83207326

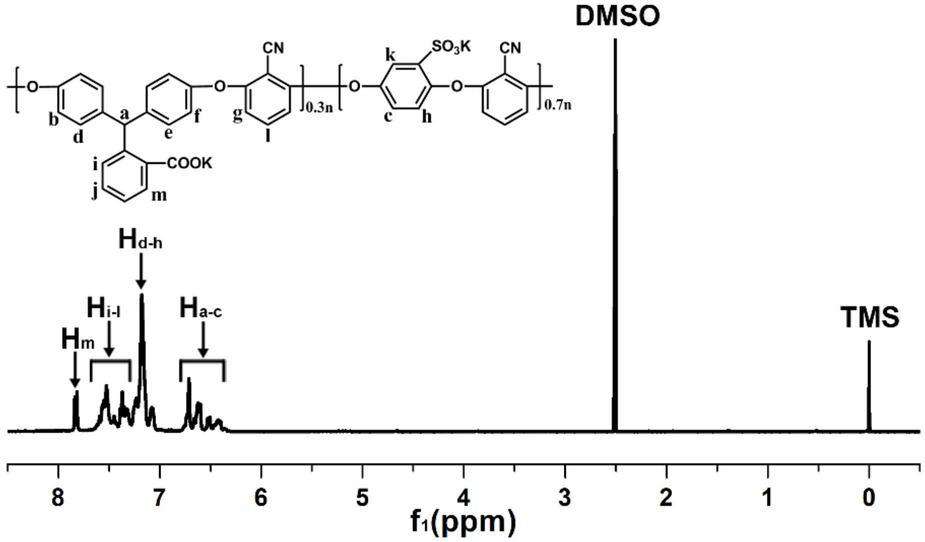

Figure S1. The 1H NMR of SPEN.

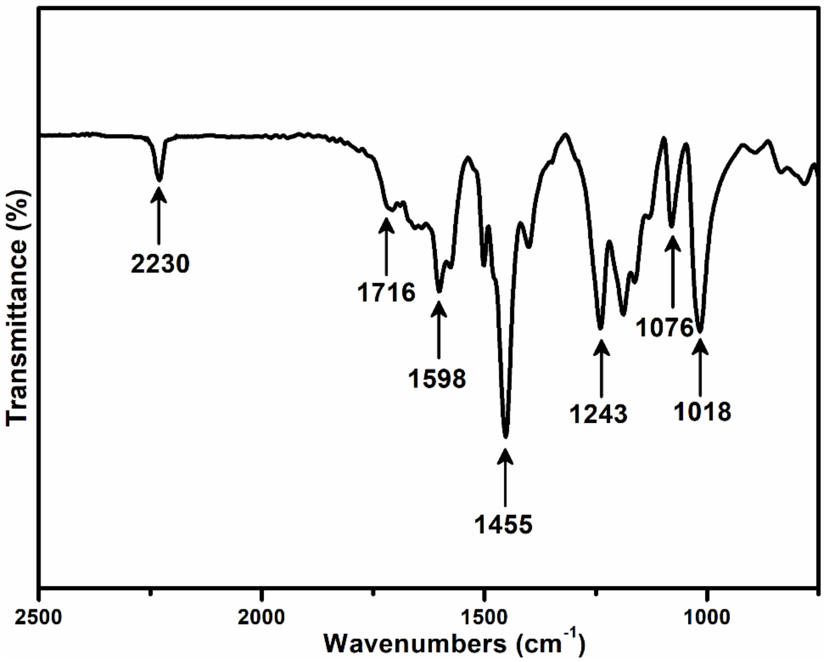

Figure S2. The FTIR of SPEN.

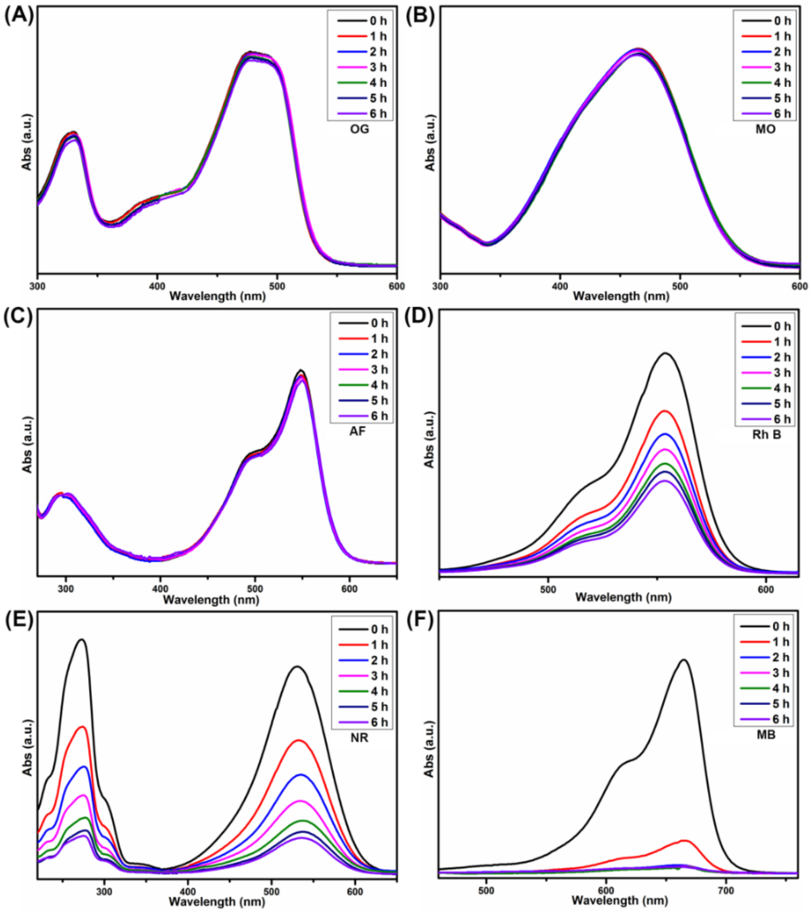

Figure S3. The UV-Vis spectra of different dyes: orange G (A), methyl orange (B), acid fuchsin (C) , rhodamine B (D), neutral red (E) and methylene blue (F) adsorption onto SPEN-Al at specific time intervals, respectively.

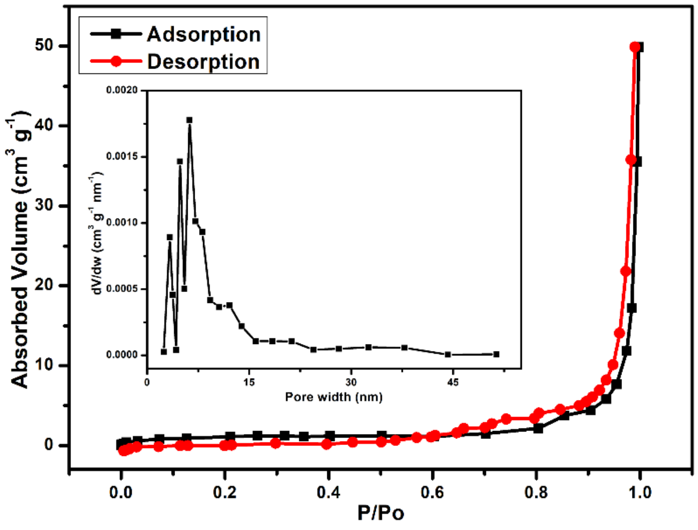

Figure S4. Nitrogen adsorption–desorption isotherm for the SPEN-Al adsorbent.
